# Spherical Lignin-Derived Activated Carbons for the Adsorption of Phenol from Aqueous Media

**DOI:** 10.3390/molecules29050960

**Published:** 2024-02-22

**Authors:** Piotr Łątka, Bazyli Olszański, Magdalena Żurowska, Marek Dębosz, Anna Rokicińska, Piotr Kuśtrowski

**Affiliations:** 1 Faculty of Chemistry, Jagiellonian University, Gronostajowa 2, 30-387 Kraków, Poland; piotr.latka@uj.edu.pl (P.Ł.); magda.zurowska@doctoral.uj.edu.pl (M.Ż.); marek.debosz@uj.edu.pl (M.D.); anna.rokicinska@uj.edu.pl (A.R.); 2 Doctoral School of Exact and Natural Sciences, Jagiellonian University, St. Łojasiewicza 11, 30-348 Kraków, Poland

**Keywords:** lignocellulosic biomass, lignin-derived activated carbons, chemical activation, phenolic compounds, adsorption

## Abstract

In this work, a synthesis and activation path, which enabled the preparation of spherical activated carbon from a lignin precursor, characterized by high adsorption capacity in the removal of phenolic compounds from water, was successfully developed. Two industrial by-products, i.e., Kraft lignin and sodium lignosulfonate, were used to form spherical nanometric lignin grains using pH and solvent shift methods. The obtained materials became precursors to form porous activated carbons via chemical activation (using K_2_CO_3_ or ZnCl_2_ as activating agents) and carbonization (in the temperature range of 600–900 °C). The thermal stabilization step at 250 °C was necessary to ensure the sphericity of the grains during high-temperature heat treatment. The study investigated the influence of the type of chemical activator used, its quantity, and the method of introduction into the lignin precursor, along with the carbonization temperature, on various characteristics including morphology (examined by scanning electron microscopy), the degree of graphitization (evaluated by powder X-ray diffraction), the porosity (assessed using low-temperature N_2_ adsorption), and the surface composition (analyzed with X-ray photoelectron spectroscopy) of the produced carbons. Finally, the carbon materials were tested as adsorbents for removing phenol from an aqueous solution. A conspicuous impact of microporosity and a degree of graphitization on the performance of the investigated adsorbents was found.

## 1. Introduction

Access to clean air and safe water is crucial for the existence of life on Earth. Meanwhile, the rapid development of industries, population growth, and continuous improvement in the standard of living have resulted in an increased demand for usable water, and have also had a devastating impact on the water resources through the release of large amounts of contaminants. An important group of emitted pollutants are organic compounds dissolved in water, including phenols and their derivatives, which can bioaccumulate in the environment [1,2,3]. It should be remembered that these substances are formed naturally as a result of the decomposition of organic matter by microorganisms or the metabolic processes of many species of plants and fungi [4]. However, currently, the chemical industry has become a substantial source of phenol emissions, where these compounds are widely used in the production of plastics, paints, dyes, and phenolic resins. Phenolic compounds are also intermediates for the production of pesticides, herbicides, bactericides, and fungicides, all commonly used as plant protection agents. Furthermore, both food industries and households contribute to the increasing concentration of these substances in groundwater, rivers, and water reservoirs. The presence of phenolic compounds in drinking water has a detrimental impact on human and animal health [5,6]. Therefore, efforts have been made to minimize the content of these harmful pollutants by developing appropriate elimination methods, including (photo)catalytic oxidation [7,8,9,10], biodegradation [11,12,13], and membrane technologies [14,15]. Particularly significant benefits, however, are achieved through the use of adsorbents, which enable the quick, easy, and effective removal of phenols, for example, in batch or flow systems. This process enables the recovery of adsorbed compounds through desorption, coupled with the regeneration of the adsorbent bed.

Activated carbons, produced from almost any natural organic carbon-enriched material, are highly effective adsorbents of phenols [16,17,18,19,20,21]. The accumulation of phenolic compound molecules appearing at the surface of activated carbon during the adsorption process usually results from the interaction of the π electrons of the phenyl ring with the π electrons of the graphene layers in the structure of the carbon material or the formation of hydrogen bonds between surface groups, containing heteroatoms and hydroxyl groups in phenol molecules. The formation of donor–acceptor complexes between surface electron-donor groups and the acidic (electrophilic) aromatic ring is also possible [22,23].

In addition to carbonization, the preparation of activated carbons often includes activation processes, leading to the formation and development of porosity, thus directly influencing adsorption capacity [24,25]. Typically, the previously carbonized carbon material is activated, but the activation process can be also combined with carbonization in order to obtain an active adsorbent in one step. Activation is often based on mixing a raw material with an activating agent (e.g., H_3_PO_4_, ZnCl_2_, KOH, K_2_CO_3_) [26,27,28]. As a result, much higher porosities of carbon materials compared to non-activated carbons are generated. The above described procedure is called chemical activation. Alternatively, physical activation is also used, which involves increasing the porosity of carbon via interaction with air or another gasifying agent (e.g., CO_2_ and/or water vapor). The adsorption capacity of activated carbon towards a specific type of adsorbate changes significantly, depending on its surface composition which is determined by the type and distribution of functional groups. Therefore, for activated carbons with comparable textural properties, which are produced using various methods or subjected to other activation, modification, or pre-carbonization processes, completely different adsorption capacities can be found.

An important raw material for the production of activated carbon may be lignocellulosic biomass, consisting mainly of cellulose, hemicellulose, and lignin, produced by plants in an amount of approximately 181.5 billion tons per year [29]. The least valuable component of this raw material is lignin. This is a burdensome waste product, formed during the production of cellulose pulp in the pulp and paper industry [30]. Due to the fact that lignin is a three-dimensional amorphous polymer which is mainly composed of phenyl propane units, its chemical composition varies depending on the plant raw material used, which in turn significantly limits the possibilities of its wider use [31,32,33]. The lignin produced during the processing of wood into paper, isolated using the Kraft process (based on the hot leaching of wood chips with an aqueous mixture of sodium base and sodium sulfide, leading to the breaking of bonds in lignocellulosic biomass) is most often burned to cover the energy demand of the entire process [34,35]. Only a small amount (2–5%) [34,36] is utilized for the production of lignin-based wood adhesives [37,38], lamination-coatings [39], biobased polymers [32], biofuels [40,41], antioxidants [42,43], UV-blocking agents [44], and carbon materials [45].

The use of lignin as a biopolymer with high carbon content seems to be the most economical path for the production of activated carbons [46]. Hayashi et al. [47] used black liquor from the Kraft process as a source of lignin, which was mixed with water and various activators (K_2_CO_3_, Na_2_CO_3_, KOH, NaOH, ZnCl_2_, H_3_PO_4_). The highest surface areas (approx. 2000 m^2^·g^−1^) were observed for the carbonized K_2_CO_3_-activated precursors. Rowlandson et al. [48] used lignin from the organosolv process, which was then thermally treated at various temperatures in an inert gas without the use of chemical activators. The produced activated carbons exhibited surface areas as high as 1400 m^2^·g^−1^, and showcased favorable performances in hydrogen storage (1.9 wt% at 77 K, 1 bar). Utilizing low sulfur acid hydrotropic lignin activated with H_3_PO_4_ at 450 °C resulted in activated carbons with expanded surface areas (up to 2000 m^2^·g^−1^). These activated carbons proved to be effective adsorbents of organic compounds [49]. The adsorption capacity for the Congo red dye reached ca. 65 mg·g^−1^, whereas the methylene blue reached −535 mg·g^−1^.

In the presented work, a synthesis and activation method was developed to obtain spherical lignin-derived carbons, characterized by high adsorption capacity in the removal of phenolic compounds from aqueous solutions. The inspiration came from the paper by Lievonen et al. [35], who used tetrahydrofuran (THF) as a solvent for lignin, which precipitated when introduced into water. However, the described procedure is not applicable to other lignins, and it is necessary to regulate the pH during precipitation. On the other hand, the most important challenge was the activation of lignin-derived carbon in the phenol adsorption process, while maintaining the spherical shape of the grains. Extensive research was conducted on the influence of both lignin precursors and the conditions of chemical activation (including the type, quantity, and method of introducing the ZnCl_2_ or K_2_CO_3_ activator), as well as thermal treatment (involving stabilization and carbonization) on various aspects such as sphericity, textural parameters, surface composition, and consequently, the adsorption capacity of the resulting carbon materials.

## 2. Results and Discussion

### 2.1. Synthesis of Spherical Lignin and Corresponding Carbon Particles

**Figure 1:**
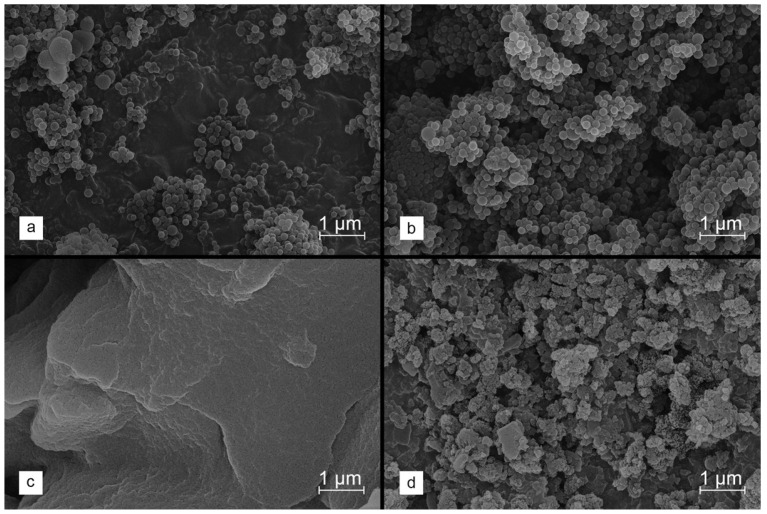
SEM images collected for lignin precipitated from solutions containing Kraft lignin (**a**,**b**) or sodium lignosulfonate (**c**,**d**). The synthesis conditions are summarized in Table 1.

In the first stage of the research, the formation of spherical lignin nanograins from two different, commercially available precursors—Kraft lignin and sodium lignosulfonate—was investigated. The use of methods was based on time-consuming procedures, such as solvothermal synthesis [50], as well as those involving expensive surfactants [51], and this was therefore abandoned. Instead, the facile synthesis strategy, based on pH shifts (shifting the pH of the mixture with dissolved lignin to a range where it is insoluble) and solvent shifts (adding a solvent that does not dissolve lignin into a mixture with dissolved lignin) was applied [52]. It should be noted that Kraft lignin is soluble in some organic solvents and in alkali, while water is a decent solvent for lignosulfonate. Therefore, the solvent shift method was tested first by introducing the Kraft lignin, dissolved in THF, into the excess water. However, SEM images revealed an aggregation of the formed lignin spheres (Figure 1a). More homogeneous and separated lignin nanospheres were obtained by lowering the pH of the final solution by adding 2M HCl (Figure 1b).

On the other hand, similar conditions did not result in the precipitation of dissolved lignosulfonate. It proved necessary to shift the pH using 2 M HCl. However, the processed lignin created structures that were severely aggregated (Figure 1c). Slightly greater sphericity of the lignosulfonate-derived lignin material was achieved by the initial modifications, for example, by acylation (Figure 1d), to reduce the lignin solubility at low pH levels. Nevertheless, these treatments did not bring satisfactory results. Hence, it was finally decided to use the Kraft lignin as a carbon precursor in order to obtain the expected lignin spheres for further research.

**Figure 2:**
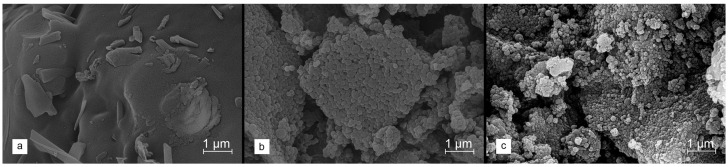
SEM images collected for spherical lignin precursor after (**a**) direct carbonization at 800 °C (heating rate of 1 °C·min^−1^), (**b**) stabilization at 250 °C (heating rate of 0.05 °C·min^−1^), and (**c**) carbonization at 800 °C (heating rate of 1 °C·min^−1^) preceded by stabilization at 250 °C (heating rate of 0.05 °C·min^−1^).

Spherical lignin nanoparticles produced in path b were subsequently carbonized. The SEM image shown in Figure 2a discloses that direct high-temperature carbonization at 800 °C leads to the sintering of the grains and a total loss of their sphericity. This effect is most likely related to the phase transformation of lignin. The glass transition temperature of lignin depends on many factors, including moisture content, the degree of branching, and molecular weight, but is typically found in the range of 110–235 °C [53]. Therefore, it was proposed to introduce an additional stage of pre-carbonization of the lignin precursor via a very gradual increase in temperature, reaching around 250 °C (a temperature ramp of 0.05 °C·min^−1^), and then maintaining that for the next 2 h [54,55]. Such stabilization of the lignin precursor allowed for the retaining of its original morphology, therefore obtaining the spherical grains of the corresponding carbon material (Figure 2b,c).

### 2.2. The Effect of the Chemical Activation of Lignin-Derived Carbons

#### 2.2.1. Morphology

**Figure 3:**
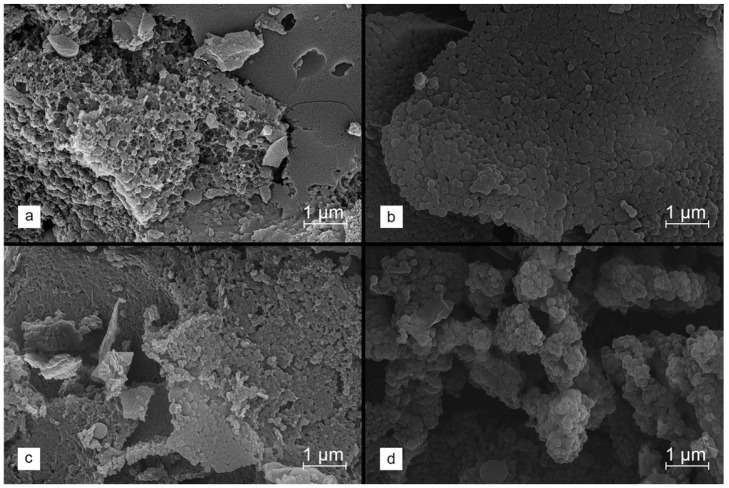
SEM images collected for stabilized lignin, chemically activated with 2.5 eq of ZnCl_2_ ((**a**)—WET method, (**b**)—DRY method) and K_2_CO_3_ ((**c**)—WET method, (**d**)—DRY method), and carbonized at 800 °C.

The effect on the properties of the formed carbons when using salts for chemical activation (i.e., ZnCl_2_ and K_2_CO_3_) was extensively studied. Considering the glass transition observed for the lignin polymer chains at approx. 250 °C, stabilized lignin was then selected for modification. As confirmed by the SEM micrographs compiled in Figure 3a–d, for such selected materials, a different degree of the preservation of spherical morphology and the aggregation of grains is observed, depending on the modification path.

In the case of the WET method, regardless of the type of salt used, a clear degradation of the spherical structure of lignin nanoparticles is visible; however, the application of ZnCl_2_ leads to the greatest changes. On the other hand, the dry incorporation of the salt allows for the better protection of the morphology of the starting material. For ZnCl_2,_ there is little sintering of the grains at the junction of lignin spheres observed, whereas the similar effect is not found in the K_2_CO_3_-activated material.

The positive effect of maintaining spherical morphology during activation with K_2_CO_3_ directed further research towards the use of this modifier. Different carbon materials were prepared using various carbonization temperatures (from 600 to 900 °C for constant K_2_CO_3_ content −2.5 eq), as well as varying ratios of K_2_CO_3_/carbon precursor (1.5 eq, 2.5 eq, 3.0 eq and 4.0 eq at the selected carbonization temperature of 800 °C), using both DRY and WET methods. In the case of the DRY method, raising the carbonization temperature to 900 °C results in the complete burning of the carbon material. On the other hand, it is possible to carbonize the sample at such a high temperature after the WET modification. However, it was found that the morphology of the carbon precursor is disturbed already at the stage of introducing salt from the aqueous solution, which is then fixed in the carbonization process. The use of the WET method results in the partial disintegration of spherical grains across all materials, and the sintering of grains into larger aggregates, in contrast to the DRY method, in which the grain morphology is retained.

**Figure 4:**
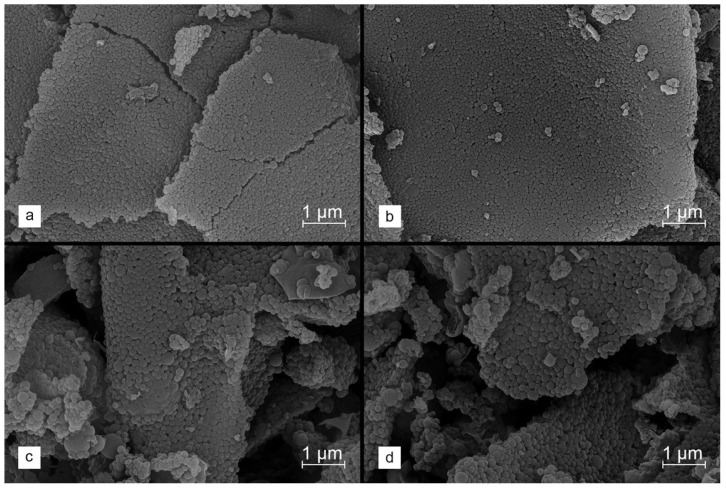
SEM images collected for stabilized lignin chemically activated with K_2_CO_3_ using the DRY method at salt to carbon precursor ratios of (**a**) 1.5 eq, (**b**) 2.5 eq, (**c**) 3.0 eq and (**d**) 4.0 eq and carbonized at 800 °C.

Figure 4a–d shows the SEM images for the carbon materials obtained using different amounts of the K_2_CO_3_ activator, introduced by the DRY method. It is observed that the activated carbons produced using lower salt/precursor ratios (1.5 eq and 2.5 eq) retain a morphology consisting of homogeneous and separated spheres. With increasing amounts of activating salt (3.0 eq and 4.0 eq), a tendency for the sintering of lignin-derived carbon grains is detected, resulting in a higher content of larger, non-uniform particles with undefined shapes across the entire volume of the material. It should therefore be stated that, when using larger amounts of the activating agent, its dispersion throughout the entire volume of the modified material begins to play a very important role in the carbonization process. An uneven distribution of the activator causes more intensive degradation of the material, which ultimately leads to the loss of the sphericality of the grains. However, even in the materials synthesized using the higher K_2_CO_3_ contents, a significant fraction of spherical-shaped lignin-derived carbon particles is still observed.

#### 2.2.2. Porosity and Graphitization

The porosity of the synthesized carbons was studied using low-temperature N_2_ adsorption measurements. The BET and Langmuir models revealed specific surface areas (S_BET_ and S_Langmuir_, respectively), while the total pore volumes (V_total_), mesopore volumes (V_meso_), and micropore volumes (S_micro_) were determined using the single-point, BJH, and t-plot methods, respectively. The textural parameters of the studied materials are shown in Table 2.

It is clearly visible that the proposed chemical activation allows for a huge increase in the porosity of the corresponding carbon materials. Potassium compounds (K_2_CO_3_ and KOH) have already been recognized as the most effective chemical activators for generating porosity in lignin-derived activated carbons, regardless of the type of precursor [47,56]. The activity of K_2_CO_3_ is related to a cycle of high-temperature transformations as follows:

$$\begin{array}{c} K_{2}{CO}_{3}\to K_{2}O+{CO}_{2}\\ {CO}_{2}+C\to 2CO\\ K_{2}{CO}_{3}+2C\to 2K+3CO\\ C+K_{2}O\to 2K+CO \end{array}$$

Both CO_x_ and very reactive K, intercalated into a carbon structure [57], are responsible for the formation of hierarchical porosity in the final carbons.

In this study, unactivated lignin, carbonized at 800 °C, creates activated carbon with S_BET_ = 187 m^2^·g^−1^ (S_Langmuir_ = 273 m^2^·g^−1^) and V_total_ = 0.181 cm^3^·g^−1^ (with contribution of ca. 41% of micropores). Depending on the nature of the precursor used during activation (L, LS or LS_C), different developments of porosity occur. Generally, the less processed the lignin substrate, the greater the susceptibility to generating pores using K_2_CO_3_. Therefore, for the least processed precursor, i.e., unstabilized lignin L, an over ninefold increase in the S_BET_ and S_Langmuir_ and a sevenfold increase in the V_total_ are achieved. In turn, in the case of the LS_C precursor, these changes are lower, being fivefold and threefold, respectively. It is worth noting, however, that the chemical activation of LS_C and LS leads to a relatively greater increase in the share of micropores (63–66% of total porosity) in the final carbon when compared to L (only 33%).

After the carbonization of stabilized lignin at 800 °C, chemically activated with various amounts of K_2_CO_3_, some changes in specific surface areas and pore volumes are observed. The higher content of the activating agent, regardless of the modification method used (DRY or WET), results in a noticeable increase in the values of textural parameters. For example, within a series of carbon materials obtained using the DRY modification, the use of 1.5 eq K_2_CO_3_ gives S_BET_ = 1457 m^2^·g^−1^ (S_Langmuir_ = 2129 m^2^·g^−1^) and V_total_ = 1.045 cm^3^·g^−1^ (V_micro_ = 0.612 cm^3^·g^−1^), while increasing the amount of the chemical activator to 4.0 eq leads to S_BET_ = 1807 m^2^·g^−1^ (S_Langmuir_ = 2681 m^2^·g^−1^) and V_total_ = 1.267 cm^3^·g^−1^ (V_micro_ = 0.630 cm^3^·g^−1^). Therefore, the use of a huge excess of K_2_CO_3_ in relation to the modified carbon precursor allows the increase of the specific surface area of the final product by 24–26% and its total pore volume by 21%. Interestingly, excess K_2_CO_3_ favors a change in the porosity profile towards the formation of larger pores, with a decreasing share of micropores. However, for the WET series, the described correlations are less conspicuous.

The analysis of the results of N_2_ adsorption reveals that the carbonization temperature has a much greater impact on the porosity of the formed carbons. A linear increase in the values of the specific surface area and total pore volume is observed with the thermal treatment temperature rising from 600 °C to 900 °C, irrespective of the modification method employed. It is worth noting that the DRY activation of stabilized lignin results in its complete decomposition during carbonization at 900 °C; therefore, Table 2 does not include textural data for this sample. For example, within the WET series, there is an increase in S_BET_ = 771 m^2^·g^−1^ (S_Langmuir_ = 1116 m^2^·g^−1^) and V_total_ = 0.419 cm^3^·g^−1^ (V_micro_ = 0.365 cm^3^·g^−1^) for the material carbonized at 600 °C to S_BET_ = 1851 m^2^·g^−1^ (S_Langmuir_ = 2732 m^2^·g^−1^) and V_total_ = 1.162 cm^3^·g^−1^ (V_micro_ = 0.677 cm^3^·g^−1^), following thermal treatment at 900 °C. At the same time, the contribution of microporosity in the total porosity changes from 87% to 58%. Similar relationships are also presented by a series of materials obtained by the DRY modification, but have also been observed in the previous studies for Kraft lignin or corn straw lignin, activated with potassium compounds [47,58]. It should therefore be stated that the increase in carbonization temperature favors the formation of the hierarchical porosity of the lignin-derived activated carbons.

**Figure 5:**
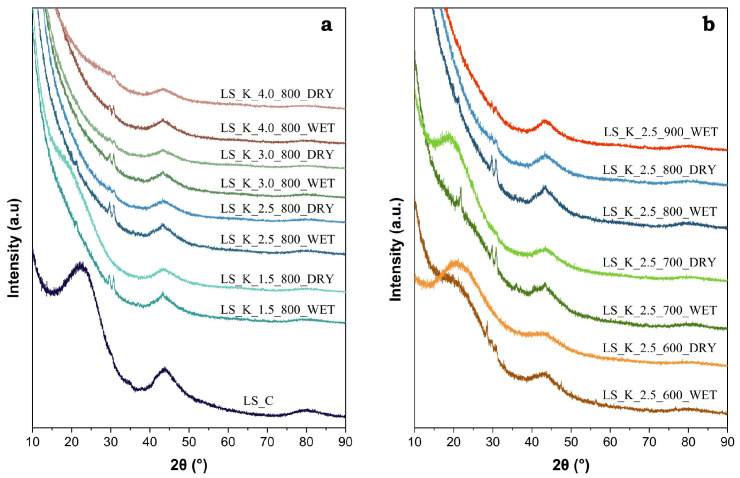
XRD patterns of selected activated carbons obtained on the basis of lignin: (**a**) LS modified with various amounts of K_2_CO_3_ (1.5–4.0 eq) and carbonized at 800 °C, and (**b**) LS modified with 2.5 eq of K_2_CO_3_ and carbonized at different temperatures (600–900 °C).

The degree of the graphitization of the studied materials was analyzed based on the XRD results. Figure 5 shows the recorded diffractograms for a series of carbons obtained by the modification of LS, with various amounts of K_2_CO_3_ after carbonization at 800 °C (panel a) and with a fixed amount of activating agent (2.5 eq), by varying the thermal treatment temperature within the range of 600–900 °C (panel b).

The presence of a broad diffraction peak (002) in the range of 15–30° 2θ is typical for the amorphous structure of activated carbon. However, it is worth paying attention to the low and wide maximum (100) at ca. 43°, which indicates the pre-formation of the graphite structure [59]. The intensity of this peak increases significantly with the carbonization temperature and confirms the expected changes in the degree of graphitization. On the other hand, in the case of a series of carbon materials obtained at the same temperature after modification with various amounts of K_2_CO_3_, the half-widths of the diffraction peak (100) remain unchanged (Figure 5b). Therefore, the activator does not have a significant impact on the achieved degree of graphitization.

#### 2.2.3. Surface Composition

**Figure 6:**
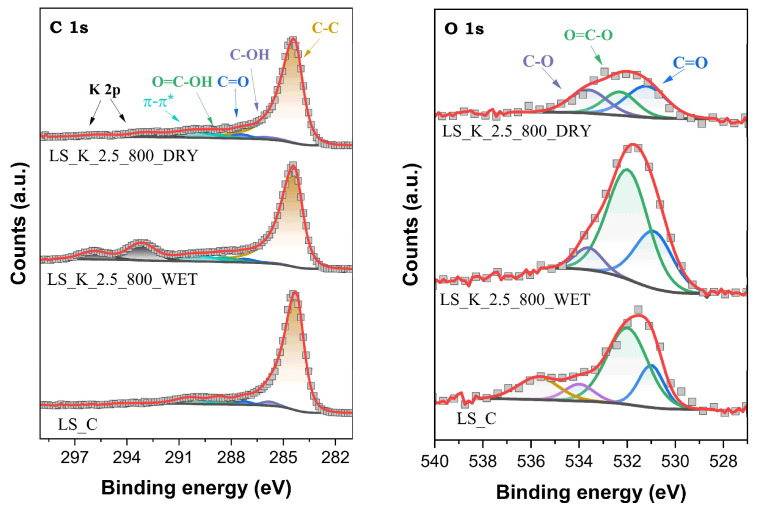
XPS C 1s and O 1s spectra for selected activated carbons obtained on the basis of lignin.

Kraft lignin used to produce activated carbons is a natural raw material; therefore, it contains a significant amount of various ingredients that remain in the material after processing. The surface analysis of LS_C by XPS revealed the presence on its surface, in addition to elemental carbon and significant amounts of oxygen, as well as, in much smaller concentrations, sulfur, nitrogen, and selected inorganic components (mainly Na). The XPS C 1s and O 1s spectra for unactivated carbon LS_C and two materials activated under the selected conditions are shown as examples in Figure 6.

In the case of XPS O 1s spectra, the presence of three main components is identified at binding energies (E_b_) of 531.0 ± 0.2, 532.1 ± 0.2, and 533.8 ± 0.2 eV, assigned to oxygen atoms in C=O, O=C–O, and C–O surface functionalities, respectively. Additionally, for LS_C carbon, the appearance of one more component at 535.7 eV, as attributed to photoelectron emission from H_2_O molecules, suggests the presence of moisture in this sample. In turn, in the XPS C 1s spectra, five components are found at 284.4 eV (C sp^2^ and sp^3^), 285.9 ± 0.1 eV (C–OH), 287.4 ± 0.2 eV (C=O), 288.6 ± 0.2 eV (COOH), and 290.4 ± 0.2 eV (π–π*) [60,61]. In the XPS C 1s spectra of lignin-derived carbons after activation, above the E_b_ values characteristic of C, additional signals appear from the emission of photoelectrons from K atoms, remaining in small amounts on the surface after treatment with K_2_CO_3_. The K 2p_3/2_ peak is observed at 293.1 ± 0.1 eV, and is accompanied by a spaced spin-orbit K 2p_1/2_ component with Δ = ~2.8 eV [62].

A complete analysis of the shares of the individual forms of surface species containing C and O atoms is presented in Table 3. It is clearly seen that the activation of stabilized lignin with K_2_CO_3_ favors the formation of oxygen-containing groups. Their total content on the surface of the carbonized material reaches even 13.4 at.% (LS_K_2.5_700_WET). It should be emphasized that both the content and distribution of surface oxygen concentrations in the created carbon materials do not differ significantly from the analogues described in the literature. For example, KOH-activated pine lignin and poplar lignin carbonized at 700 °C showed a total surface O content of 7.6 and 11.5 at.%, respectively [63]. Generally, generating larger amounts of superficial oxygen functionalities is facilitated by higher amounts of K_2_CO_3_ being introduced into the modified lignin, using both the WET method and lower carbonization temperature. It is also worth noting that C=O groups are slightly more likely to form on the surface of lignin-derived activated carbon after activation in the WET path when compared to carboxyl groups.

#### 2.2.4. Adsorption Capacity

**Figure 7:**
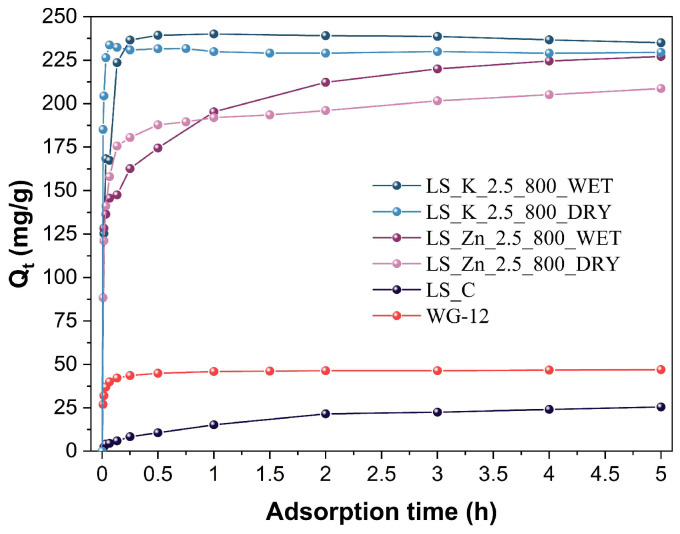
Kinetic curves of phenol adsorption on unactivated or activated (with 2.5 eq of K_2_CO_3_ or ZnCl_2_) lignin-derived carbons after carbonization at 800 °C, compared to commercial WG-12 activated carbon.

The developed carbon materials were tested as adsorbents of phenol from an aqueous solution. The adsorption tests were performed at room temperature. First, the kinetics of the process was studied in the presence of representative LS_K_2.5_800_WET and LS_K_2.5_800_DRY adsorbents, as well as analogously modified and carbonized LS_Zn_2.5_800_WET and LS_Zn_2.5_800_DRY materials, which were chemically activated with ZnCl_2_. The obtained results are presented in Figure 7, along with the kinetic data collected for non-activated carbon (LS_C) and commercial WG-12 activated carbon.

The very large application potential of carbonized, activated lignins is clearly visible, given their substantially higher adsorption capacities when compared to the reference adsorbents. However, a much better performance is observed for the samples activated with K_2_CO_3_ than ZnCl_2_. Therefore, the former materials were further analyzed to determine an influence of the amount of the activator used, the modification method, and the carbonization temperature on the observed adsorption capacities.

Table 4 demonstrates the parameters collected for the fitting of two kinetic models—pseudo-first order and pseudo-second order—to the experimental points measured for the samples of the LS_K_x_y_DRY and LS_K_x_y_WET (where x—eq. of used salt, y—carbonization temperature) series, modified with a K_2_CO_3_ amount varying from 1.5 to 4.0 eq, carbonized at 800 °C, and with a fixed K_2_CO_3_ content (2.5 eq) subjected to thermal treated at different temperatures.

The coefficients of determination (R^2^) in the case of both models are relatively high; however, slightly better fits are obtained using the pseudo-first order model. As can be seen in Figure 7, the complete saturation of the carbon material with the adsorbate is very fast and usually does not exceed 0.5 h. Therefore, the values of the adsorption rate constant (k′) have values within the narrow range of 0.8–2.6 min^−1^. No clear correlations are found between the k′ values and the activation and carbonization conditions.

From an application point of view, in addition to the process rate, the adsorption capacities obtained for the investigated activated carbons are even more important. When using different amounts of the K_2_CO_3_ modifier, after carbonization at 800 °C, activated carbons show similar Q_e_ values in the range of 219–238 mg·g^−1^. The exception are the samples synthesized by the DRY method, using the lowest and highest concentrations of K_2_CO_3_ (i.e., 1.5 eq and 4.0 eq), for which Q_e_ drops to 148 and 197 mg·g^−1^, respectively. The Q_e_ values for the carbons carbonized at different temperatures exhibit much clearer trends. Within the LS_K_2.5_y_WET series, Q_e_ increases from 144 mg·g^−1^ for the sample heated at 600 °C, to 240 mg·g^−1^ for the material carbonized at 900 °C. An even greater change is observed in the LS_K_2.5_y_DRY series, where Q_e_ increases from 76 to 231 mg·g^−1^ when the carbonization temperature raises from 600 °C to 800 °C.

**Figure 8:**
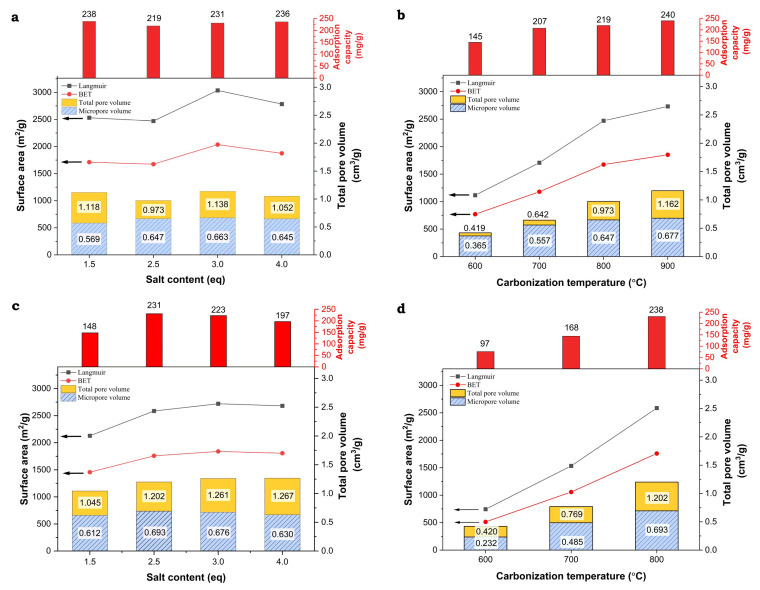
Correlations between structural parameters determined by N_2_ adsorption and phenol adsorption capacities for the lignin-derived activated carbons ((**a**)—LS_K_x_800_WET, (**b**)—LS_K_2.5_y_WET, (**c**)—LS_K_x_800_DRY, and (**d**)—LS_K_2.5_y_DRY).

When analyzing the above trends, one can notice a strong correlation between the adsorption capacity of the obtained carbon materials and their porosity, which, as shown in Table 2, is clearly determined by the carbonization temperature. To make it easier to recognize these correlations, the values of the textural parameters and Q_e_ are comparatively presented in Figure 8.

**Figure 9:**
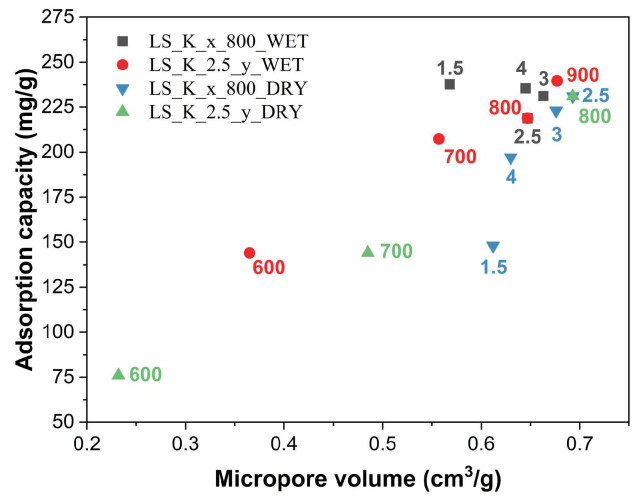
Influence of micropore volume on adsorption capacities of the lignin-derived activated carbons.

Generally, with an increase in the value of the textual parameters of the studied carbon materials, higher adsorption capacities of phenol are observed. However, upon analyzing these trends in more detail, it can be seen that the increase in adsorption capacity is mainly determined by the microporosity generated in the activated carbons (Figure 9). Its formation is favored by a higher carbonization temperature, as well as by the appropriate amount and method of introducing the activating agent (K_2_CO_3_), however to a lesser extent. Furthermore, it must not be forgotten that an increase in the carbonization temperature also promotes progressive graphitization. The participation of graphitic domains in the phenol adsorption process, characterized by the interaction of the π electrons of the phenolic ring with the π electrons of the graphene layers, may be of significance. However, there is no relevant impact of the presence of surface oxygen-containing functional groups on the adsorption capacities.

**Figure 10:**
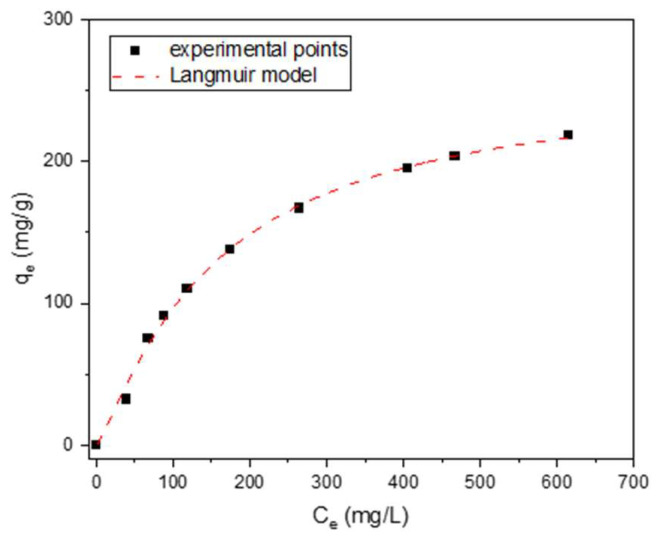
Isotherm of phenol adsorption measured for LS_K_2.5_800_DRY at room temperature.

For one of the most effective lignin-derived adsorbents, LS_K_2.5_800_DRY, a phenol adsorption isotherm was collected at room temperature. The experimental points shown in Figure 10 are very well described by the Langmuir model (R^2^ = 0.9998) as follows:

$$Q_{e}=\frac{Q_{max}K_{L}C_{e}}{1+K_{L}C_{e}}$$

where Q_max_ (mg/g) is the maximum adsorption capacity, and K_L_ (L/mg) is the Langmuir constant as it related to the energy of adsorption. It is worth noting that the Q_max_ achieved in the presence of this carbon adsorbent is 260 mg·g^−1^. This value is higher than most of the reported efficiencies of activated carbons in phenol removal, e.g., commercial materials (including WG-12 studied in this work) and chemically activated carbons, obtained from Kraft lignin (74–137 mg·g^−1^) [18].

Taking into account such high adsorption capacities, as well as the availability of raw material, simplicity, and low production costs, developed spherical lignin-derived activated carbons are excellent candidates for commercial applications. The feature of sphericity seems to be particularly important in this context, as it ensures the greater repeatability of the properties of individual grains, along with the increased availability of gaseous and liquid phase components into pores, as well as their flexibility in forming various bed geometries.

## 3. Materials and Methods

### 3.1. Chemicals and Materials

Kraft lignin (Sigma-Aldrich, Poznań, Poland); sodium lignosulfonate (TCI, Tokyo, Japan); tetrahydrofuran (THF, Sigma-Aldrich, ≥99.0%); hydrochloric acid (HCl, solution 37%, Honeywell, Warsaw, Poland); acetic acid (Chempur, Piekary Śląskie, Poland, ≥99.5%); *N*,*N*-dimethylformamide (DMF, Chempur, ≥99.8%); ethyl acetate (Chempur, ≥99.5%); ethanol (Chempur, ≥96.0%); acetone (Chempur, ≥99.0%); zinc chloride (ZnCl_2_, Sigma-Aldrich, ≥97.0%); potassium carbonate (K_2_CO_3_, Sigma-Aldrich, ≥99.0%); phenol (Chempur, ≥98.0%); nitrogen (5.2, Air Products, Warszawa, Poland); commercial activated carbon WG-12 (Gryfskand, Gryfino, Poland).

### 3.2. Synthesis

The pre-preparation of lignin precursors to obtain spherical nanoparticles was based on methods adopted from the literature [35,64]. Three materials were used as lignin precursors—Kraft lignin, sodium lignosulfonate, and acylated sodium lignosulfonate. While the first two materials were used as commercially available, the latter was synthesized by the acylation of sodium lignosulfonate. For this purpose, 10 g of sodium lignosulfonate was dissolved in 100 mL of DMF and 100 mL of THF, and the resulting mixture was then combined with 50 mL of acetic acid mixed with 50 mL of THF. The temperature was increased to 70 °C, and the mixture was left stirring for 24 h. After that, the solvents were removed in a rotary evaporator. The obtained material was rinsed sequentially with ethyl acetate, ethanol, and acetone, to then be finally dried at 60 °C.

Furthermore, 5 g of the lignin precursor was dissolved in 75 mL of THF and 25 mL of water at room temperature under vigorous stirring. The resulting mixture was introduced dropwise into 340 mL of deionized water in a 1 L flask at room temperature. Following this, 10 mL of 2M HCl was then added slowly in the selected cases (paths b and d, presented in Table 1). In the case of sodium lignosulfonate (path c, shown in Table 1), the addition of different amounts of water and acid was required to the precipitate of the lignin material (i.e., 5 g of the lignin precursor was dissolved in 75 mL of THF and 25 mL of water, and added to 200 mL of deionized water, together with 200 mL of 2M HCl). The change of the solution color from dark brown to light milky brown was observed. After the completion of this synthesis step, THF was removed under a reduced pressure. The prepared sample was centrifuged (5000 rpm) using an MPW-352 centrifuge (MPW, Warsaw, Poland) for 300 min, and dried at 60 °C overnight. The material was then finely ground using an agate mortar and subjected to further drying at 60 °C for 24 h. Subsequently, lignin nanoparticles were pre-carbonized in a tube furnace at an air atmosphere at 250 °C for 2 h (with a temperature ramp of 0.05 °C·min^−1^, preventing the collapse of the spherical structure).

Finally, the synthesized lignin nanoparticles were carbonized in a tube furnace under a nitrogen flow (60 mL·min^−1^), optionally in the presence of ZnCl_2_ or K_2_CO_3_, to produce the corresponding carbon materials. Furthermore, 2.5 eq of the salt was introduced using two different methods. In the DRY method, both the salt and carbon precursor were mixed to be homogenized and then carbonized. Alternatively, in the WET approach, the appropriate amount of salt was dissolved in a small amount of water, and the carbon precursor was placed in the stirred solution overnight. After this, the material was dried at 60 °C and carbonized for 4 h at a temperature selected from the range of 600–900 °C (with a temperature ramp of 1 °C·min^−1^). Carbonization at 800 °C was additionally carried out with various salt equivalents (e.g., 1.5 eq, 3.0 eq and 4.0 eq). The amount of salt and the carbonization temperature used during the synthesis of a given carbon material were coded in a sample name, which is constructed as follows: [lignin precursor, where L-unstabilized lignin, LS-stabilized lignin, LS_C-stabilized lignin after carbonization]_[activator used, K for K_2_CO_3_ and Zn for ZnCl_2_]_[salt equivalent-1.5 eq, 2.5 eq, 3.0 eq or 4.0 eq]_[carbonization temperature, value used in °C]_[method used for salt introduction, DRY or WET]. After cooling, the resulting activated carbon was placed in an excess of water (250 mL of H_2_O per 1 g of a carbon material) and stirred at room temperature for 24 h. Afterwards, the carbon material was filtered, washed with 1M HCl, deionized water, and acetone, and finally dried at 60 °C to a constant mass.

### 3.3. Characterization

The structural ordering of the carbon samples was monitored using X-ray powder diffraction (XRD). The XRD patterns were collected on a Bruker D2 Phaser instrument (Bruker, Billerica, MA, USA), using Cu Kα radiation (λ = 1.54184 Å) and a LYNXEYE detector within a 2θ range of 10–90° at a step of 0.02°.

The textural properties of the materials were studied using N_2_ adsorption at −196 °C. The adsorption–desorption isotherms were measured using a Micromeritics ASAP 2020 sorptometer (Micrometrics, Norcross, GA, USA). A sample was initially outgassed at 250 °C for 6 h under vacuum conditions. Specific surface areas were calculated using the BET and Langmuir models. Single point adsorption at p/p_0_ → 1 was used to determine the total pore volumes. The micropore and mesopore volumes were obtained using the t-plot model (multipoint fitting in p/p_0_ = 0.1–0.3) and the Barrett–Joyner–Halenda (BJH) model (assuming a pore diameter range of 2–50 nm), respectively.

The morphology was investigated by scanning electron microscopy (SEM) imaging, using an Apreo 2 S LoVac field emission scanning electron microscope (Thermo Fisher Scientific, Waltham, MA, USA). A sample was mounted on a sticky carbon disc and coated with a gold layer. A secondary electrons (SE) signal was used for observation.

Surface analyses were carried out using X-ray photoelectron spectroscopy (XPS) in a system constructed by Prevac. XPS spectra were collected using a monochromatized aluminum source Al Kα (E = 1486.6 eV) and a hemispherical analyzer (VG SCIENTA R3000, Newburyport, MA, USA). The binding energy scale for the conductive carbon samples was calibrated by referring to a position of Au 4f (E_b_ = 84.0 eV). Both the Shirley background and fitting with the mixed function of Gauss and Lorentz (GL = 30) (with one exception of the main C 1s peak fitted with an asymmetric Lorentzian (LA) line shape) were used during the interpretation of the spectra in the CasaXPS software (Version 2.3.25PR1.0).

### 3.4. Adsorption Tests

The carbonized lignin nanoparticles were tested as adsorbents in the removal of phenol from an aqueous solution. In a typical test, 200 mg of a carbon material was added to 500 mL of the phenol solution with a concentration of 200 mg·L^−1^, kept in a thermostated flask (22.5 ± 0.2 °C), and mixed with a magnetic stirrer (stirring rate = 200 rpm). For the determination of adsorption isotherms, the concentration of phenol in the solution varied from 40 mg·L^−1^ to 615 mg·L^−1^. The phenol concentrations in the samples withdrawn from the studied aqueous solution were determined using the spectrophotometric method. The spectra were collected in an Evolution 220 (Thermo Scientific, Waltham, MA, USA) dual-beam UV–Vis spectrometer, equipped with a xenon lamp within a λ range of 200–400 nm, a resolution of 0.1 nm, and a scanning time of 60 min^−1^.

The efficiency of phenol adsorption was analyzed for the carbonized lignin materials calculating adsorption capacity (Q_t_), according to the following formula:

$$Q_{t}=\frac{V\times (C_{0}-C_{t})}{m}$$

where V is the solution volume, m—the adsorbent amount (g), C_0_—the initial phenol concentration in the solution (mg·L^−1^), and C_t_—the phenol concentration in the solution after time t (mg·L^−1^).

The pseudo-first order (PFO) and pseudo-second order (PSO) models were used to describe the kinetics of phenol adsorption on the studied activated carbons. The applied equations in non-linear and dimensionless forms are presented in Table 5.

Within this table, $Q_{e}$ is the amount of phenol adsorbed at equilibrium (mg·g^−1^), $k_{ad}^{\prime }$ is the pseudo-first order adsorption rate constant (min^−1^), $k_{ad}^{\prime \prime }$ is the pseudo-second order adsorption rate constant (g·mg^−1^·min^−1^), and t is the time of the adsorption process (min).

## 4. Conclusions

An effective and facile synthesis of spherical nanometric lignin particles, based on precipitation from an aqueous solution, was developed. It was shown that a selection of lignin starting materials with an appropriate solubility in water was extremely important. The Kraft lignin grains retained their sphericity after carbonization, even up to 800 °C. Furthermore, chemical activation, especially with K_2_CO_3_, resulted in the development of high specific surface area (S_BET_ up to 2036 m^2^ g^−1^) and hierarchical pore systems, characterized by a dominant share of micropores (V_micro_ up to 0.693 cm^3^∙g^−1^), effectively interconnected by the presence of wider mesopores (V_meso_ up to 0.618 cm^3^∙g^−1^). As a result, the resulting activated carbons exhibited a high adsorption capacity (even higher than 250 mg∙g^−1^) in removing phenol from aqueous solutions. The adsorption capacity was particularly favored by the presence of micropores and graphitic domains, created in the carbonized lignin.

## Figures and Tables

**Table 1:** Conditions used during the precipitation of spherical lignin particles.

Sample Code	Lignin Precursor	Initial Solution	Final Solution	
		THF/H_2_O (*v*/*v*)	THF/H_2_O (*v*/*v*)	HCl Conc. (mol·L^−1^)
a	Kraft	3/1	15/73	0
b			15/73	0.045
c	Sodium lignosulfonate		3/17	0.800
d	Acylated sodium lignosulfonate		15/73	0.045

**Table 2:** The textural parameters of the selected activated carbons obtained on the basis of lignin.

Sample Code	S_BET_ (m^2^·g^−1^)	S_Langmuir_ (m^2^·g^−1^)	V_total_ (cm^3^·g^−1^)	V_meso_ (cm^3^·g^−1^)	V_micro_ (cm^3^·g^−1^)
No activation, carbonization at 800 °C					
LS_C	187	273	0.181	0.088	0.075
Activation with K_2_CO_3_ (various contents), carbonization at 800 °C					
LS_K_1.5_800_WET	1711	2531	1.118	0.528	0.568
LS_K_1.5_800_DRY	1457	2129	1.045	0.255	0.612
LS_K_2.5_800_WET	1674	2471	0.973	0.274	0.647
L_K_2.5_800_WET	1763	2622	1.358	0.618	0.449
LS_C_K_2.5_800_WET	920	1348	0.592	0.189	0.374
LS_K_2.5_800_DRY	1759	2586	1.202	0.292	0.693
LS_K_3.0_800_WET	2036	3036	1.138	0.418	0.663
LS_K_3.0_800_DRY	1840	2720	1.261	0.352	0.676
LS_K_4.0_800_WET	1874	2784	1.052	0.349	0.645
LS_K_4.0_800_DRY	1807	2681	1.267	0.431	0.630
Activation with K_2_CO_3_ (2.5 eq), carbonization at various temperatures					
LS_K_2.5_600_WET	771	1116	0.419	0.041	0.365
LS_K_2.5_600_DRY	514	745	0.420	0.072	0.232
LS_K_2.5_700_WET	1179	1707	0.642	0.065	0.557
LS_K_2.5_700_DRY	1058	1533	0.769	0.119	0.485
LS_K_2.5_900_WET	1851	2732	1.162	0.460	0.677

**Table 3:** Concentrations of O and C on the surface of selected lignin-derived activated carbons determined by XPS.

Sample Code	Content of O Species (at.%)				Content of C Species (at.%)					
	C=O	O=C–O	C–O	Total	sp^2^, sp^3^	C–OH	C=O	COOH	π–π*	Total
No activation, carbonization at 800 °C										
LS_C	1.49	4.37	0.69	6.60	76.16	1.71	1.54	2.06	4.11	85.60
Activation with K_2_CO_3_ (various contents), carbonization at 800 °C										
LS_K_1.5_800_WET	2.65	3.78	1.43	7.90	72.04	1.32	2.24	1.96	7.37	84.90
LS_K_1.5_800_DRY	0.78	1.85	0.79	3.40	86.18	0.78	0.78	1.55	7.28	96.60
LS_K_2.5_800_WET	2.65	5.52	0.75	8.90	62.51	0.61	1.69	2.60	5.96	73.40
LS_K_2.5_800_DRY	1.94	1.20	1.35	4.50	79.83	1.87	2.28	2.25	6.88	93.10
LS_K_3.0_800_WET	5.93	4.48	1.93	12.30	60.20	1.36	4.14	2.13	5.93	73.80
LS_K_3.0_800_DRY	1.14	2.51	1.36	5.00	83.86	1.50	1.14	1.43	5.29	93.20
LS_K_4.0_800_WET	4.00	5.28	1.00	10.30	65.80	0.95	4.13	2.66	5.09	78.60
LS_K_4.0_800_DRY	1.47	7.76	1.58	10.80	73.40	1.44	1.34	3.91	4.16	84.30
Activation with K_2_CO_3_ (2.5 eq), carbonization at various temperatures										
LS_K_2.5_600_WET	5.05	3.80	1.09	9.90	72.55	1.09	4.75	1.75	4.09	84.20
LS_K_2.5_600_DRY	6.09	4.10	2.09	12.30	69.04	2.17	2.46	2.52	4.37	80.60
LS_K_2.5_700_WET	8.80	2.95	1.61	13.40	61.46	1.25	2.96	1.54	6.04	73.30
LS_K_2.5_700_DRY	3.03	3.28	1.49	7.80	77.04	1.77	3.13	1.57	4.71	88.20
LS_K_2.5_900_WET	3.44	5.24	1.06	9.70	66.40	2.85	2.00	2.40	6.64	80.30

**Table 4:** Kinetic parameters of pseudo-first-order and pseudo-second-order models for the adsorption of phenol by selected lignin-derived carbons, activated with K_2_CO_3_.

Sample Code	Pseudo-First Order			Pseudo-Second Order		
	$Q_{e}$ (mg∙g^−1^)	$k_{ad}^{\prime }$ (min^−1^)	R^2^	$Q_{e}$ (mg∙g^−1^)	$k_{ad}^{\prime \prime }$ (g∙mg^−1^∙min^−1^)	R^2^
Activation with K_2_CO_3_ (various contents), carbonization at 800 °C						
LS_K_1.5_800_WET	237.71	2.089	0.9984	239.52	0.0342	0.9963
LS_K_1.5_800_DRY	147.97	1.069	0.9997	148.94	0.0275	0.9999
LS_K_2.5_800_WET	218.88	1.163	0.9999	219.01	0.0359	0.9999
LS_K_2.5_800_DRY	231.05	2.317	0.9997	233.16	0.0348	0.9966
LS_K_3.0_800_WET	231.23	1.821	0.9996	234.00	0.0231	0.9962
LS_K_3.0_800_DRY	223.06	1.540	0.9996	225.72	0.0792	0.9999
LS_K_4.0_800_WET	235.53	1.770	0.9993	238.23	0.0234	0.9956
LS_K_4.0_800_DRY	197.05	2.644	0.9995	197.67	0.1099	0.9989
Activation with K_2_CO_3_ (2.5 eq), carbonization at various temperatures						
LS_K_2.5_600_WET	143.97	0.980	0.9883	138.01	0.0096	0.9767
LS_K_2.5_600_DRY	75.99	1.255	0.9988	76.77	0.0587	0.9993
LS_K_2.5_700_WET	207.33	1.091	0.9998	207.94	0.0573	0.9997
LS_K_2.5_700_DRY	144.11	0.777	0.9954	145.71	0.0185	0.9966
LS_K_2.5_900_WET	239.56	2.387	0.9991	242.14	0.0330	0.9946

**Table 5:** Model equations describing the kinetics of the phenol adsorption [65,66].

Kinetic Model	Non-Linear
Pseudo-first order (PFO)	$Q_{t}=Q_{e}\times \left(1-exp\left(-k_{ads}^{\prime }\times t\right)\right)$
Pseudo-second order (PSO)	$Q_{t}=\frac{k_{ads}^{\prime \prime }\times Q_{e}^{2}\times t}{1+k_{ads}^{\prime \prime }+Q_{e}\times t}$

## Data Availability

The raw data supporting the conclusions of this article will be made available by the authors on request.
